# Effects of low concentrations of fatty acids on *Escherichia coli* depend on the kind of culture medium and incubation temperature

**DOI:** 10.3389/abp.2025.15672

**Published:** 2025-11-17

**Authors:** Barbara Stencel, Monika Zielenkiewicz, Łukasz Grabowski

**Affiliations:** 1 Faculty of Medicine, Medical University of Gdansk, Gdansk, Poland; 2 Department of Molecular Biology, University of Gdansk, Gdansk, Poland

**Keywords:** bacterial growth, cultivation conditions, *Escherichia coli*, fatty acids, antibacterialproperties

## Abstract

Fatty acids play important, yet different roles in bacterial physiology, specifically their growth, either stimulating or inhibiting this parameter which are of biotechnological importance. Here, we present results showing to what degree short- and medium-chain fatty acids (butyric acid (butanoic acid, C4:0); caproic acid (hexanoic acid, C6:0); caprylic acid (octanoic acid, C8:0)), used at relatively low concentrations (in a range of μg/mL, contrary to previously reported mg/mL which revealed inhibitory effects on bacterial growth) affect growth of *Escherichia coli* K-12 (MG1655 laboratory strain) depending on various conditions. In rich medium (LB) positive effects of all tested fatty acids on *E. coli* growth were observed, while temperature of incubation (growth at 25 °C and 37 °C was assessed) modulated these effects. In contrast, a slight but significant growth inhibition by fatty acids was observed in a minimal medium (M9) supplemented with glucose. Nonetheless, in minimal medium containing acetate, the effects of these compounds varied, being either positive or negative depending on their concentrations. No measurable bacterial growth was observed in the case of the presence of any tested fatty acids when primary carbon source (glucose or acetate) was removed from a minimal medium before addition of butyric acid, caproic acid or caprylic acid. Our results indicated that effects of low concentrations of fatty acids on *E. coli* cells depend on growth conditions of bacterial cultures. This may be of biotechnological importance, especially for modulating *E. coli* growth by using different compositions of media and incubation temperatures.

## Introduction

Fatty acids play important roles in physiology of both prokaryotic and eukaryotic organisms (de Carvalho and Caramujo, 2018). These compounds are commonly classified according to the length of their aliphatic chain; when molecules contain 6 or less carbon atoms, they are called “short-chain fatty acids,” the chains made of 8–10 carbons are characteristic for “medium-chain fatty acids,” and “long-chain fatty acids” contain 12 carbons or more. In bacteria, fatty acids are components of cell membranes, being either produced in cells or directly incorporated into bacterial membranes from external sources (van den Berg et al., 2025), and can be used either as a carbon source or for storage material production (Pavoncello et al., 2022). Furthermore, fatty acids are known for their antibacterial activities (Casillas-Vargas et al., 2021).

When fatty acids serve as carbon/energy source, their molecules must be transported into bacterial cells and then degraded in specific biochemical pathways. Short-chain and medium-chain fatty acids can enter the cell through either a free diffusion or using porins in membranes, while long-chain fatty acids require specialized importer proteins (Black and DiRusso, 2023). The degradation pathway of fatty acid is conducted through the β-oxidation cycles, the evolutionary conserved process which is best understood in *Escherichia coli* (Pavoncello et al., 2022).

Despite using fatty acids as a carbon source, various reports described antibacterial properties of these compounds (Casillas-Vargas et al., 2021). Early experiments demonstrated that caprylic acid (octanoic acid) and capric acid (decanoic acid) revealed antimicrobial properties toward *E. coli*, which was not observed for short-chain and long-chain fatty acids (Marounek et al., 2003). On the other hand, a strong inhibition of *E. coli* growth was reported for both caproic acid and caprylic acid (De Keyser et al., 2019). Further studies, based on proteomic analyses, suggested that antibacterial effects of caprylic acid were caused predominantly due to damaging of cell membranes and inducing oxidative stress (Rodríguez-Moyá and Gonzalez, 2015).

Therefore, in the light opposite effects of fatty acids on bacteria summarized above (the source of carbon and energy, and growth inhibition), we asked about conditions which might cause predominance of one of these effects, positive or negative. Determination of such a feature would be valuable for applied microbiological purposes, especially for optimizing cultivation conditions in bioreactors during biotechnological production as well as for intestinal microbiota regulation in various therapies. Since the bacterial metabolism of fatty acids and their antibacterial properties are best described for *E. coli* (see above), we have employed the widely used *E. coli* K-12 strain MG1655. As antibacterial effects of short-chain and medium-chain fatty acids were mostly investigated and described in previous reports, we used butyric acid (butanoic acid, C4:0), caproic acid (hexanoic acid, C6:0), and caprylic acid (octanoic acid, C8:0). However, contrary to most previous reports mentioned above, where concentrations of fatty acids were in a range of mg/mL, we focused on the use of low concentrations of these compounds, in a range of mg/mL, as the latter conditions might better reflect those occurring in mammalian intestine, as well as those easily reached during bacterial cultivation for biotechnological purposes. Moreover, different growth temperatures were tested, 37 °C and 25 °C; the former temperature reflecting natural *E. coli* habitat (mammalian gut) and standard laboratory conditions, while the latter temperature being employed for slow bacterial growth in cultures as well as occurring in an external environment, i.e., outside the mammalian body.

## Methods

### Bacterial strain and culture conditions

**FIGURE 1:**
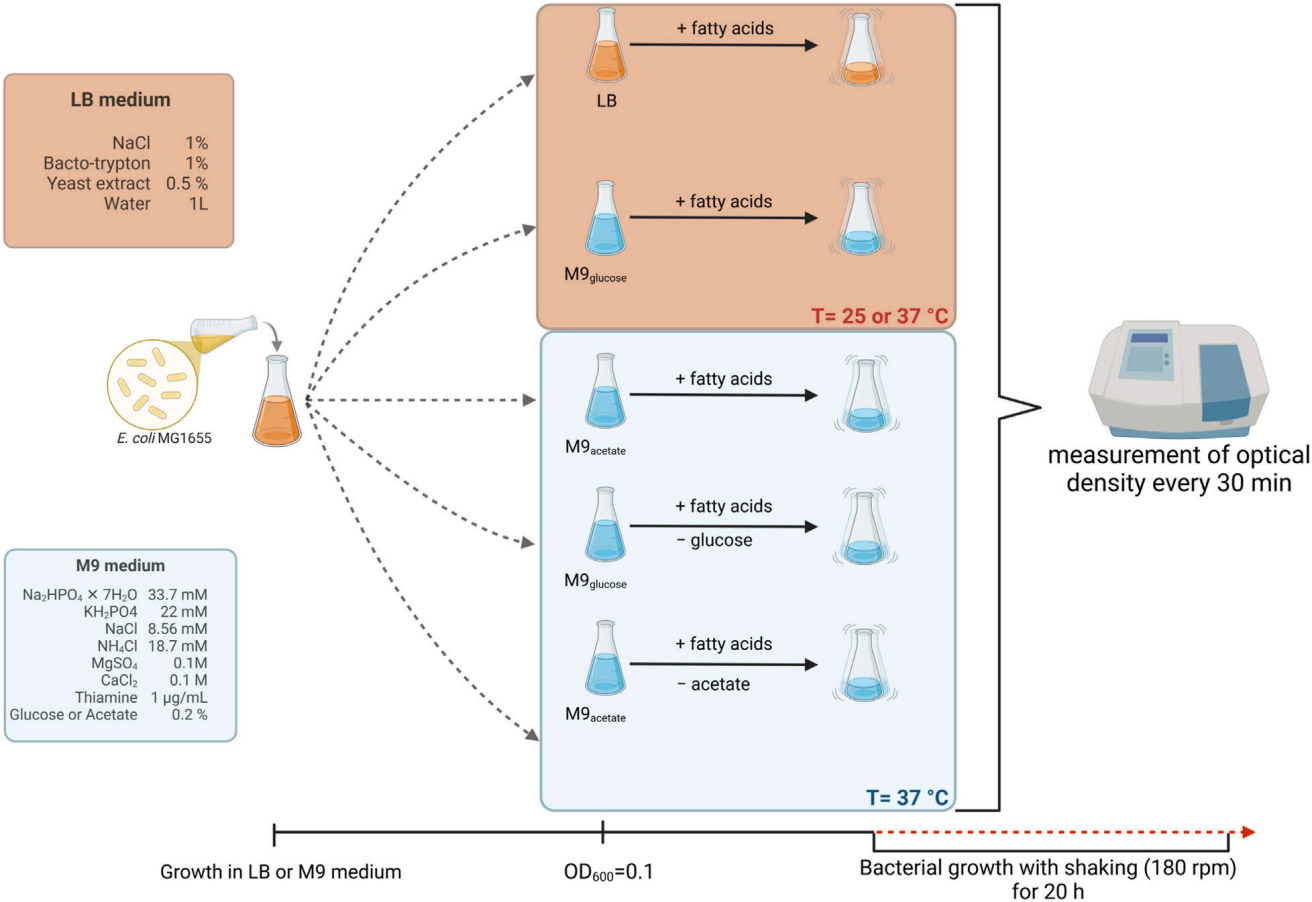
Scheme of the experimental procedures. Bacteria were cultured in indicated media (their compositions are indicated on the left side of the scheme) to OD_600_ = 0.1, and then procedures were conducted as depicted. The scheme was created using BioRender.com.

*Escherichia coli* K-12 strain MG1655 (Jensen, 1993) was used in the experiments. Bacteria were cultured at 25 °C or 37 °C in LB (BioShop, Burlington, ON, Canada) or M9 medium (BioShop, Burlington, ON, Canada). Bacteriological agar (BioShop, Burlington, ON, Canada) at a final concentration of 1% was used in solidified media (LB-agar or M9-agar). The exact compositions of the media and the scheme of the experiment are shown in Figure 1 (the scheme is also shown, in a simplified form, in Supplementary Figure S1).

### Saturated fatty acids

Three saturated fatty acids were used in the experiments: butyric acid (butanoic acid, C4:0), caproic acid (hexanoic acid, C6:0), and caprylic acid (octanoic acid, C8:0) (all purchased from Warchem, Poland). All acids were dissolved in 100% DMSO (Pol-Aura, Poland), and solutions of these acids were prepared in sterile water (Eurx, Poland) at concentrations of 16 mg/mL, 8 mg/mL, 4 mg/mL, 2 mg/mL, 1 mg/mL, 0.5 mg/mL, and 0.25 mg/mL. The final concentration of DMSO in these solutions was 0.5%. Then, these solutions were added to the medium at 1:1000 (v/v) ratio to obtain final concentrations of 16, 8, 4, 2, 1, 0.5, and 0.25 mg/mL, respectively.

### Testing effects of saturated fatty acids as an additional carbon source on the growth of *Escherichia coli*

An overnight culture of *E. coli* MG1655 was inoculated from one bacterial colony grown on LB-agar or M9-agar medium and cultured in LB or M9 medium with (180 rpm) for 24 h at 25 °C or 37 °C. Then, the overnight culture of bacteria was suspended in LB or M9 medium at a 1:100 ratio (v/v) and cultured in a water bath with shaking (180 rpm) at 25 °C or 37 °C.

At OD_600_ = 0.1 (measured by using SmartSpecPLUS, BIO-RAD, CA, USA) bacteria were loaded to the wells of a 96-well plate and appropriate concentrations of butyric, caproic or caprylic acid were added, and cells were cultured with shaking at 25 °C or 37 °C for 20 h, with measuring of OD_600_ every 0.5 h (measured by using Varioskan Lux, Waltham, MA, USA). The negative control was a bacterial culture growing in LB or M9 medium with the addition of an appropriate amount of sterile water (Eurx, Poland).

### Testing effects of saturated fatty acids as the only carbon source on the growth of *Escherichia coli*

The initial stages of the experiment were analogous to those presented in Section *Testing effects of saturated fatty acids as an additional carbon source on the growth of Escherichia coli* with some modifications. When the bacterial culture reached OD_600_ = 0.1 (measured by using SmartSpecPLUS, BIO-RAD, CA, USA), it was centrifuged at 4000 ×g for 15 min at 4 °C (Avanti JXN-26, rotor JS-13.1, Beckman Coulter, IN, USA). The supernatant was removed, and the bacterial pellet was dissolved in DPBS buffer (Gibco, Waltham, MA, USA) to eliminate residual medium and centrifuged at 4000 ×g for 15 min at 4 °C (Avanti JXN-26, rotor JS-13.1, Beckman Coulter, IN, USA). Then, the supernatant was removed and the bacterial pellet was dissolved in LB or M9 medium without a carbon source. A saturated fatty acid was added to the bacterial culture at indicated concentration and bacteria were cultured with shaking for 20 h, with measuring OD_600_ every 0.5 h (measured by using Varioskan Lux, Waltham, MA, USA). The negative control was a bacterial culture growing in M9 medium with glucose or acetate as a carbon source.

### Statistical analysis

Statistical analysis of the data was performed using IBM SPSS v.14 software. First, the normality distribution of the data was checked using the Kolomogorov-Smirnov test, followed by the Levene’s test to assess the homogeneity of variance. When both of the above assumptions were met, a one-way ANOVA with Tukey *post-hoc* test was performed. If one of these assumptions was not met, the Kruskal-Wallis test with Dunn’s *post-hoc* test was performed. Statistical significance (*p* < 0.05) is marked with an asterisk (*).

## Results

The experiments were conducted using *E. coli* K-12 strain MG1655, grown in various media (nutrient medium LB or a minimal medium M9 with different carbon sources, glucose or acetate; Figure 1) and at different temperatures (25 °C and 37 °C). Three different fatty acids were used: butyric acid (butanoic acid, C4:0), caproic acid (hexanoic acid, C6:0), and caprylic acid (octanoic acid, C8:0). These fatty acids were added to bacterial cultures either without removing the primary nutrients or carbon source (glucose or acetate), or with the replacement of glucose or acetate by the tested fatty acid. The scheme of the experiment is presented in Figure 1.

When bacteria were grown at 37 °C in LB medium supplemented with fatty acids, a stimulation of growth was observed in the case of all tested compounds (butyric acid, caproic acid, and caprylic acid) at various concentrations relative to the culture without fatty acids added to the medium (the summary of the results is shown in Table 1, while detailed results are shown in Supplementary Figures S2, S3). This bacterial growth stimulation was evident especially during the late exponential/stationary phase when *E. coli* culture densities continued to increase in the presence of fatty acids, whereas bacteria incubated in LB alone ceased to grow.

**TABLE 1:** Summary of effects of fatty acids on the growth of *E. coli* under different cultivation conditions.

Conditions^a^	Effects of fatty acids on *E. coli* growth^b^		
	Butyric acid (butanoic acid, C4:0)	Caproic acid (hexanoic acid, C6:0)	Caprylic acid (octanoic acid, C8:0)
LB, 37 °C	↑ (LE/S)	↑ (LE/S)	↑ (LE/S)
LB, 25 °C	↑ (ME)	↑ (ME)	↑ (ME)
M9 Glu, 37 °C	↓ (LE)	↓ (LE)	↓ (LE)
M9 Glu, 25 °C	↓ (ME)	↓ (ME)	↓ (ME)
M9 Ace, 37 °C	↑ (ME)	↑↓ (ME)	↑↓ (ME)
M9 Glu, 37 °C, after Glu removal	0	0	0
M9 Ace, 37 °C, after Ace removal	0	0	0

^a^ Bacteria were cultured in LB medium (LB), M9 medium with 0.2% glucose (M9 Glu) or M9 medium with 0.2% acetate (M9 Ace) at indicated temperatures. Media were supplemented with different fatty acids. In some experiments, glucose or acetate was removed from M9 before addition of fatty acids.

^b^ The effects of fatty acids on *E. coli* growth are indicated relative to that observed in corresponding media without fatty acids. Up arrow (↑) indicates growth stimulation, down arrow (↓) indicates growth inhibition, and up and down arrows (↑↓) indicate growth stimulation or inhibition depending on fatty acid concentration; zero (0) indicates no measurable bacterial growth. Abbreviations in parentheses indicate growth phases where the most pronounced effects were determined, as follows: (LE), late exponential phase; (ME), mid-exponential phase; (S), stationary phase (in this case, entering the stationary growth phase at different OD_600_ values of the culture was observed).

Stimulation of *E. coli* growth in LB medium by all tested fatty acids was also observed at 25 °C. However, differences in growth rates between cultures with and without butyric acid, caproic acid, or caprylic acid became evident earlier under these conditions, specifically during the mid-exponential phase (Table 1; Supplementary Figures S4, S5).

Contrary to the *E. coli* growth stimulation by fatty acids observed in LB medium, no growth rate increases could be detected when bacteria were cultured in the M9 minimal medium with glucose. Indeed, a small but statistically significant decrease in the growth rate was evident in the presence of butyric acid, caproic acid or caprylic acid at certain concentrations, relative to the control culture devoid of fatty acids. Such an effect was noted at both 37 °C (Table 1; Supplementary Figures S6, S7) and 25 °C (Table 1; Supplementary Figures S8, S9).

Intriguingly, when acetate was used instead of glucose as a carbon source in the M9 minimal medium, effects of fatty acids on *E. coli* growth were either positive or negative, depending on specific concentrations of these compounds and the kind of fatty acid. Nevertheless, while only stimulatory effects could be noted in the presence of butyric acid, both caproic acid or caprylic acid caused either stimulation or inhibition of the bacterial growth at various concentrations (Table 1; Supplementary Figures S10, S11). We suspect that untypical, quite a “heterogeneous,” growth of bacterial cultures under these conditions, with some temporary slowdowns and accelerations, might arise from a limited availability of acetate as a carbon source and somewhat unstable physiological conditions.

Totally different results were obtained in experiments where primary carbon sources (either glucose or acetate) were removed from the M9 minimal medium before addition of fatty acids. Under such conditions, no measurable growth of *E. coli* was observed in the presence of every tested fatty acid (butyric acid, caproic acid, or caprylic acid), irrespective of the concentration, and irrespective of the kind of the primary carbon source washed out prior to fatty acid supplementation, either glucose (Table 1; Supplementary Figures S12, S13) or acetate (Table 1; Supplementary Figures S14, S15).

## Discussion

Fatty acids play various roles in organisms, from structural functions as constituents of phospholipids occurring in cell membranes, storage materials, and molecules involved in cell signalling (de Carvalho and Caramujo, 2018). Nevertheless, in the case of bacteria, fatty acids may play a negative role, as the antibacterial effects of these compounds have been reported previously (Marounek et al., 2003; De Keyser et al., 2019; Casillas-Vargas et al., 2021). Such effects are believed to be mainly due to membrane damage and oxidative stress (Rodríguez-Moyá and Gonzalez, 2015). However, bacteria can metabolize fatty acids (Pavoncello et al., 2022), thus, they are able to use them to improve their energetic status. Therefore, it appears that there are two independent actions of fatty acids on bacterial cells, one of which is beneficial and the other is destructive. The major concern is what determines the domination of one process over another.

The strong biological activity of fatty acids is mainly represented by short-chain and medium-chain compounds (Marounek et al., 2003; De Keyser et al., 2019). Since butyric acid, caproic acid, and caprylic acid were identified as revealing the most pronounced effects on bacteria (Zavišić et al., 2024), while occurring also in human diet or produced by probiotic microorganisms, we have tested what conditions might distinguish between beneficial and deleterious effects of these fatty acids on a model bacterium, *E. coli* K-12 strain MG1655. Contrary to previous reports, where micromolar or mg/mL concentrations of fatty acids were used (Marounek et al., 2003; De Keyser et al., 2019; van den Berg et al., 2025), we focused on their low concentrations (in a range of μg/mL). We were aware that such amounts of fatty acids were perhaps too low to have strong toxic effects on bacteria, however, such conditions should be more similar to those occurring in the natural environment of *E. coli* (mammalian gut) and might be easily obtained in cultures of this bacterium conducted for biotechnological purposes.

We have demonstrated that all tested fatty acids stimulated growth of *E. coli* in LB (rich) medium, especially at the later stages. We assume that these compounds might be used as alternative carbon sources when other molecules became exhausted from the medium due to their consumption by bacterial cells. Interestingly, there were differences between the effects of fatty acids at various temperatures, as growth stimulation was observed at the earlier phase of cultivation at 25 °C (mid-exponential phase) than at 37 °C (late exponential/stationary phase). This difference might be caused by either different composition of cell membranes at various temperatures (influencing fatty acid transportation or susceptibility of membranes to fatty acid-mediated damage) or the rate of cellular metabolism and putative requirement for additional nutrients at lower temperature.

Contrary to the growth in rich medium, when minimal medium was used, the positive effect of fatty acids on *E. coli* growth was either absent (when glucose was employed as a carbon source) or less pronounced and dependent on both the kind of fatty and its concentration (when acetate was employed as a carbon source). However, drastic effects of all tested fatty acids on the bacterial growth were evident when a bacterial culture was incubated first in a minimal medium with glucose or acetate, and the carbon source was removed just before addition of a fatty acid. Under these conditions, *E. coli* growth was undetectable irrespective of both the kind of fatty acid and its concentration. Evidently such conditions could not support the use of fatty acids as a sole carbon source. Because of low concentrations of these compounds used (from 0.25 to 16 μg/mL), we assume that their strong toxic effects were rather unlikely, thus, the most probable explanation is that *E. coli* cannot employ such molecules are sole carbon sources under starvation conditions.

On the basis of results presented in this report, we conclude that effects of short-chain and medium-chain fatty acids on *E. coli* depend strongly on growth medium (rich or minimal) and temperature, with a strong effect of the presence/absence of a specific carbon source (glucose or acetate in the case of our experiments) at the time of fatty acid addition. These results might also explain some ostensible discrepancies which can be found in the literature in respect of deleterious effects of specific short- and medium-chain fatty acids on *E. coli* (compare, for example, Marounek et al., 2003; De Keyser et al., 2019). In fact, differences between results presented in various reports could arise, as least partially, from using different growth conditions of bacterial cultures. Obviously, the use of different concentrations of fatty acid is another plausible reason for obtaining ostensibly contradictory results.

Overall, our results provide a clear and methodologically solid foundation for further studies aimed at elucidating the molecular mechanisms of fatty acid action in *E. coli*. However, the present data are largely phenomenological, and the interpretation remains partly speculative, as no biochemical or molecular assays were used to confirm the proposed mechanisms. Moreover, the study was limited to the *E. coli* K-12 laboratory strain, which, while well-characterized and highly reproducible, may not fully reflect the diversity of responses among other *E. coli* strains, including environmental and clinical isolates. Differences in membrane composition, regulatory networks, or metabolic flexibility could influence how various strains respond to exogenous fatty acids.

Future research should therefore not only integrate metabolic, molecular, and omics-based approaches to comprehensively characterize how fatty acids influence bacterial physiology under different environmental conditions, but also expand the analysis to additional *E. coli* strains and other representative Gram-negative bacteria. Such comparative studies will clarify whether the observed effects are conserved across species or strain-specific, and will help to uncover broader adaptive strategies that bacteria employ when exposed to exogenous lipids.

Despite molecular mechanisms of the correlations reported in this work remain to be elucidated, we suggest that results presented here might have some implications for biotechnological and medical purposes. Namely, *E. coli* is one of the most commonly used microorganisms for production of various compound, at both small and large scales; therefore optimization of the growth of this bacterium in cultures, in flasks and in huge bioreactors, is biotechnologically important (Negrete and Shiloach, 2017). Our findings suggest that supplementation of growth media with low concentrations of specific fatty acids might modulate bacterial metabolism in a temperature-dependent manner, potentially improving biomass accumulation or production yields under certain conditions. For example, fine-tuning the type and concentration of fatty acids could be explored as a strategy to optimize *E. coli* cultures used in recombinant protein production or fermentation processes, especially when cultivation parameters such as temperature and nutrient composition are adjusted. Conversely, understanding conditions under which fatty acids inhibit growth may be useful for designing selective control strategies in mixed microbial cultures or in biocontainment approaches.

Therefore, conditions which might either improve *E. coli* growth or cause its inhibition should be considered carefully, being crucial factors influencing the efficiency of biotechnological processes. Hence, a potential usefulness of addition of low concentrations of fatty acids to growth media might be taken into account but only when a kind of medium and temperature are carefully chosen. Considering medical importance of the results reported here, it is crucial to emphasize the importance of the role of gut microbiota for human health. Since bacteria occurring in human gastrointestinal tract can influence human physiology significantly, and dysbiosis is correlated with various diseases (Chong et al., 2025; Marcari et al., 2025; Nunez et al., 2025; Trisal et al., 2025; Zhang et al., 2025), while modulating the composition of the gut microbiome (also using dietary compounds) is proposed as a potential therapeutic procedure (Cao et al., 2024; Kandari et al., 2024; Yarahmadi et al., 2024), careful consideration of providing various nutrients and additives is necessary, including assessment of possible roles of fatty acids. This also concerns prebiotics and probiotics, as it was demonstrated that various bacteria can effectively produce fatty acids (Yarahmadi et al., 2024). Thus, demonstration of various effects of fatty acids on bacterial cells depending on the growth conditions suggests that estimation of conditions occurring in human gut should be especially carefully considered when planning treatment of patients or healthy people (in the course of prevention) with nutraceuticals, prebiotics, probiotics, and other agents which might influence fatty acid composition and concentration in the gut.

## Data Availability

The original contributions presented in the study are included in the article/Supplementary Material, further inquiries can be directed to the corresponding author.
