# Supplementary material for: Effects of low concentrations of fatty acids on Escherichia coli depend on the kind of culture medium and incubation temperature
Source: Acta Biochim Pol. 2025 Nov 17;72:15672. doi: 10.3389/abp.2025.15672 (PMC12665612; doi:10.3389/abp.2025.15672)
Supplement: Supplementary file 1 [file DataSheet1.pdf]

SUPPLEMENTARY MATERIAL TO:

Effects of low concentrations of fatty acids on *Escherichia coli* depend on the kind of culture medium and temperature of incubation

Barbara Stencel, Monika Zielenkiewicz, Łukasz Grabowski

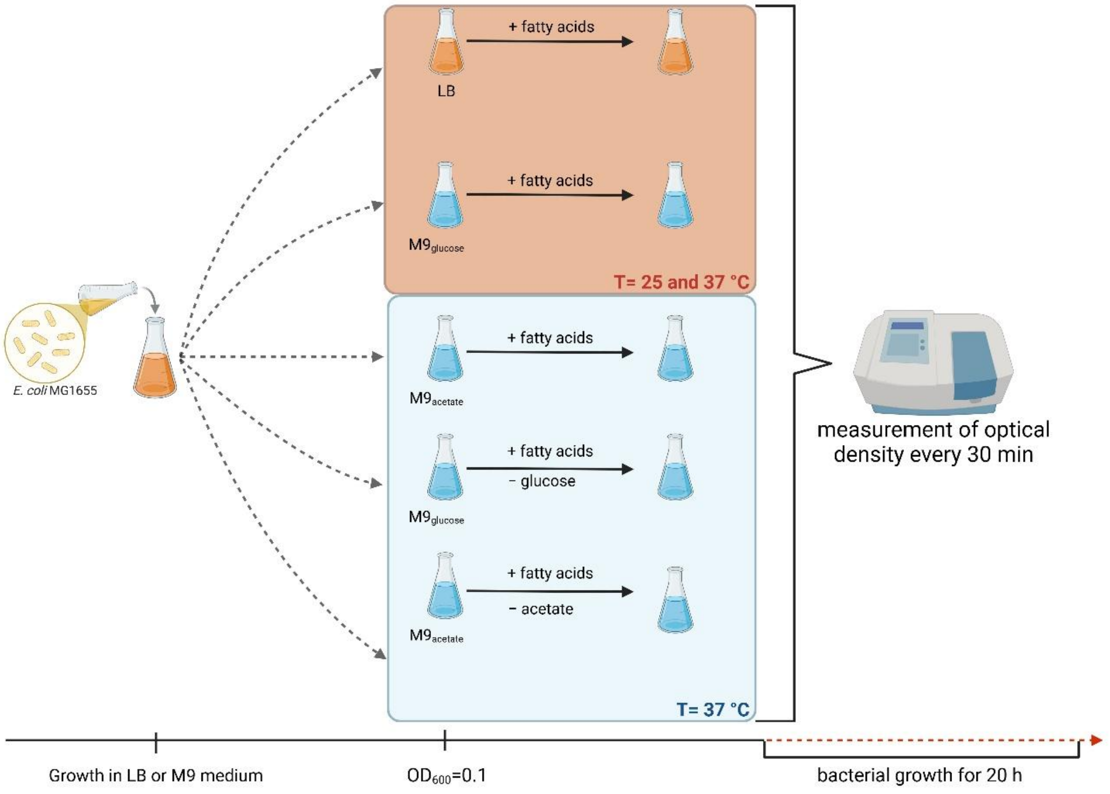

Figure S1. Scheme of the experimental procedures. Bacteria were cultured to OD_{600}=0.1, and then procedures were conducted according to the figure. The scheme was created using BioRender.com.

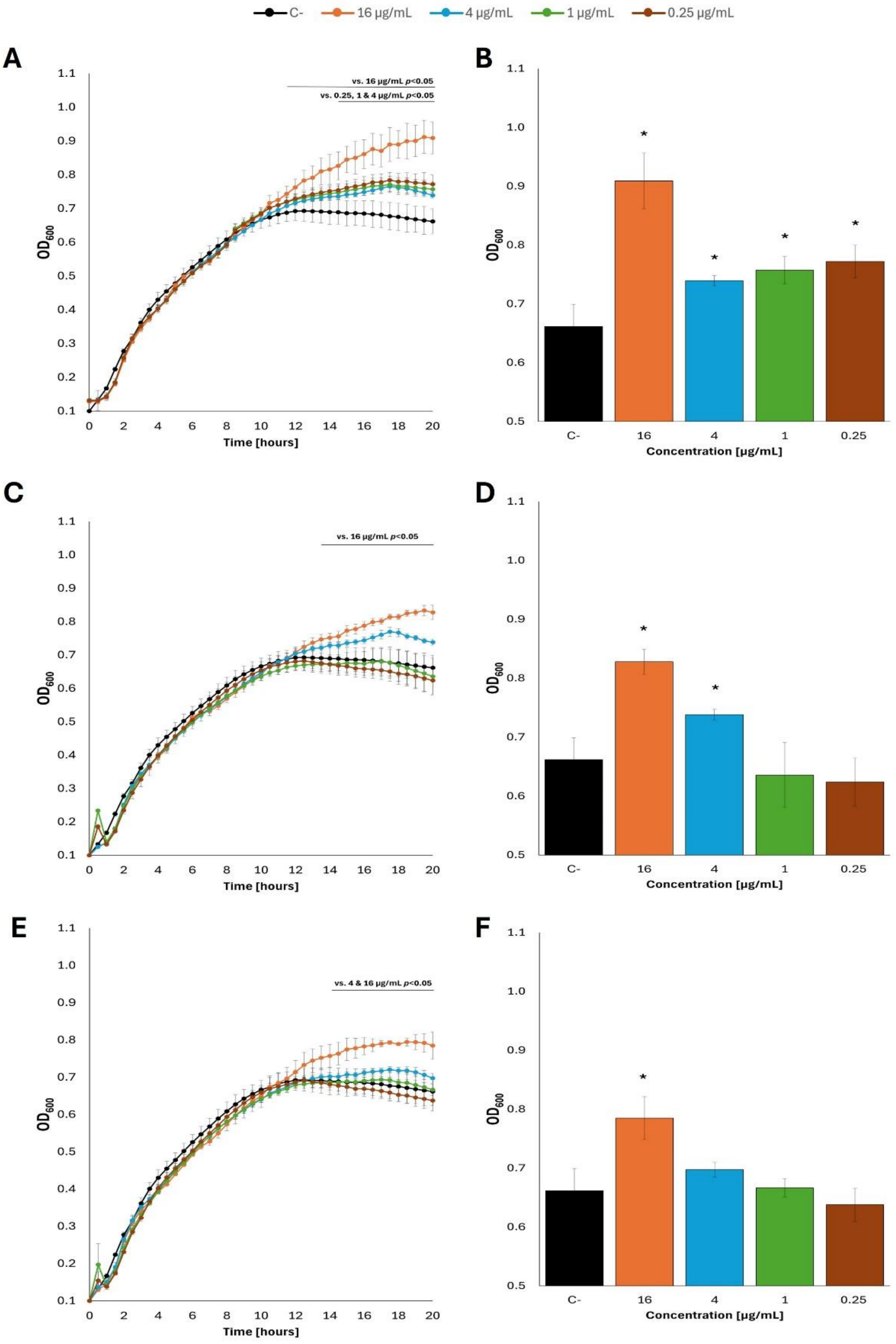

Figure S2. Growth curves of *E. coli* MG1655 with fatty acids at 37 °C in LB medium. Bacteria were cultured to OD=0.1, and then butyric acid (**A**), caproic acid (**C**) and caprylic acid (**E**) were added at appropriate concentrations. Panels B,D and F show the optical density value of the culture at the end of the experiment: butyric acid (**B**), caproic acid (**D**) and caprylic acid (**F**). Statistical significance (p<0.05) is marked with an asterisk (*).

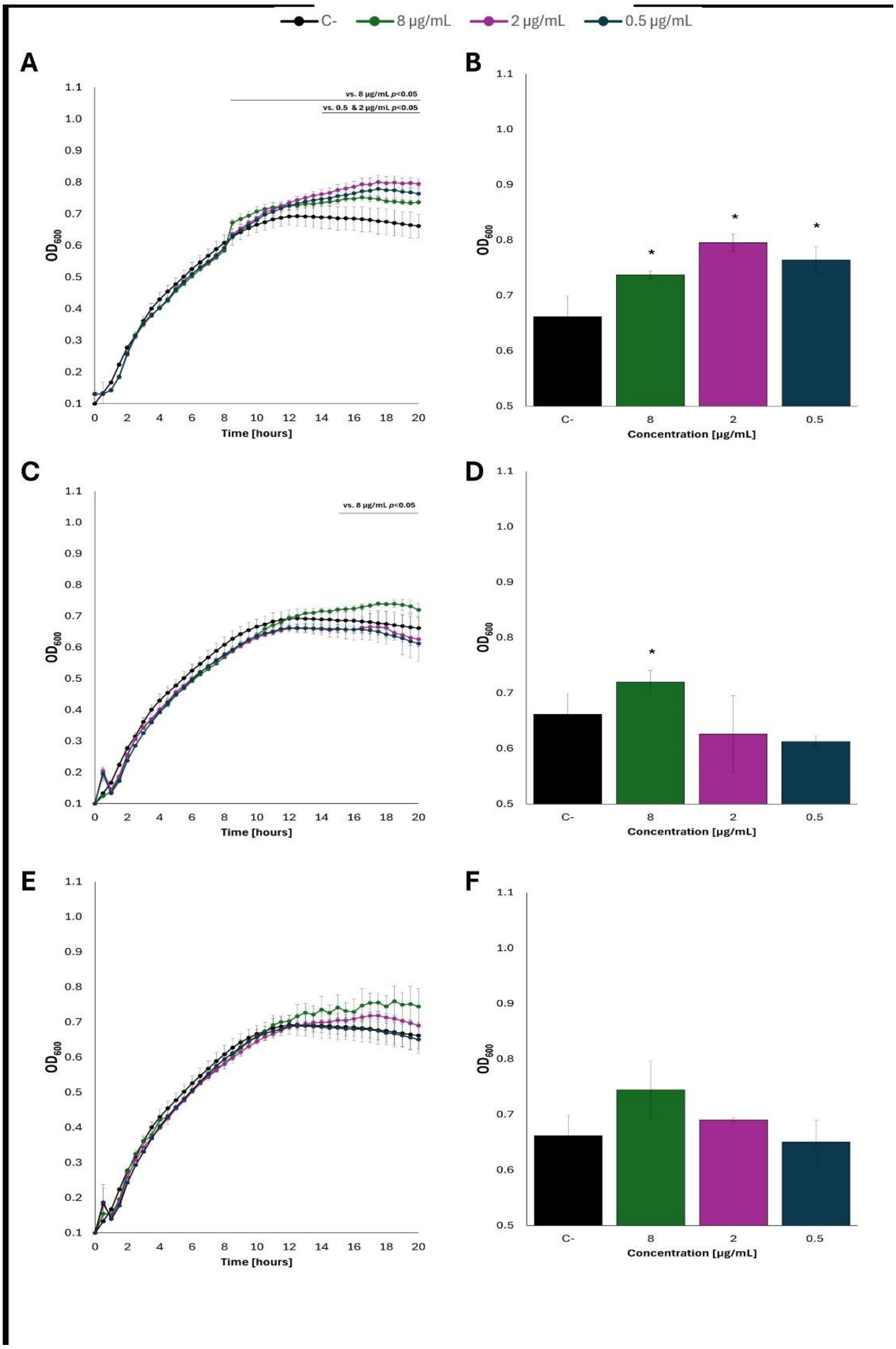

Figure S3. Growth curves of *E. coli* MG1655 with fatty acids (additional set of concentrations) at 37 °C in LB medium. Bacteria were cultured to OD=0.1, and then butyric acid (**A**), caproic acid (**C**) and caprylic acid (**E**) were added at appropriate concentrations. Panels B, D and F show the optical density value of the culture at the end of the experiment: butyric acid (**B**), caproic acid (**D**) and caprylic acid (**F**). Statistical significance ($p<0.05$) is marked with an asterisk (*).

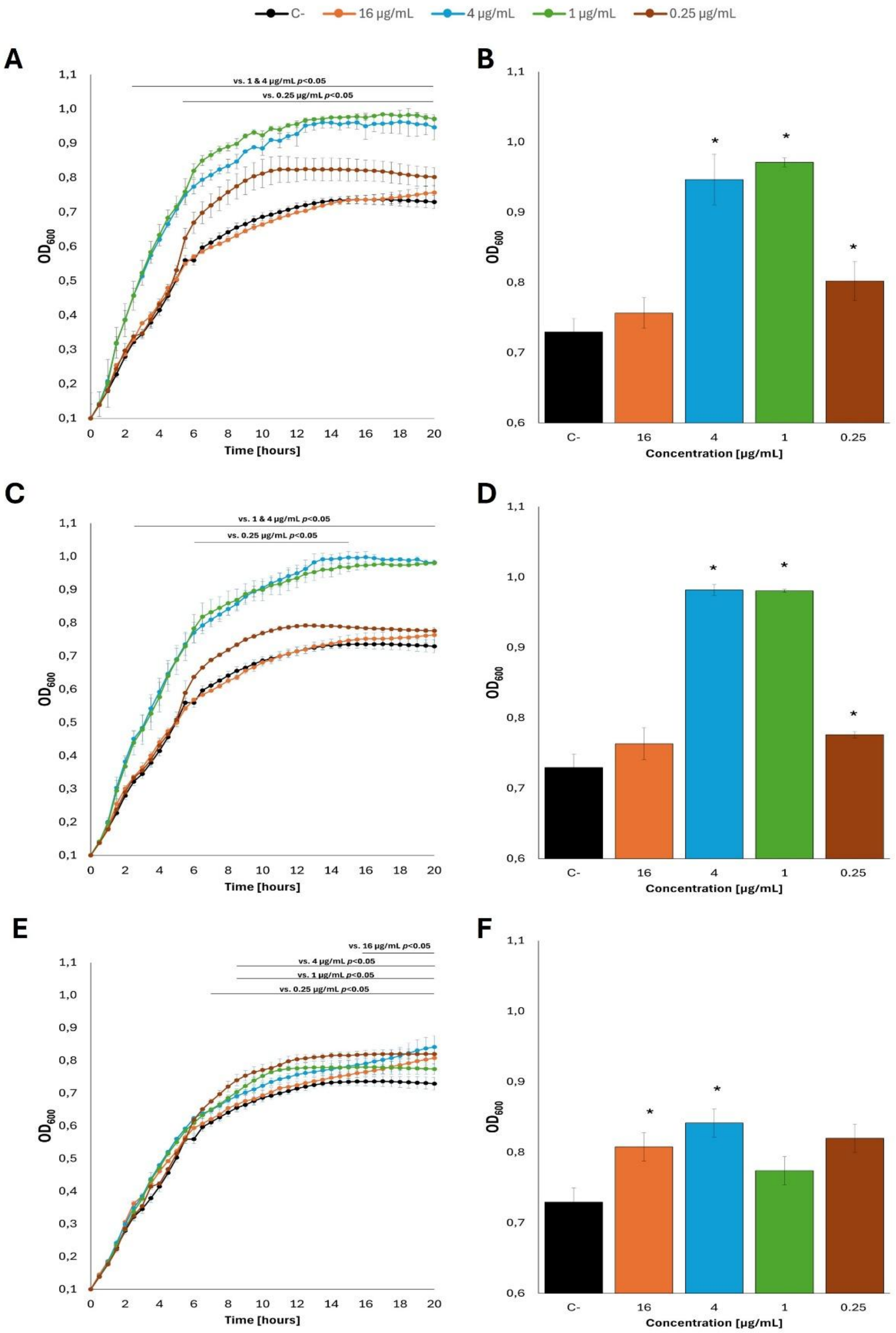

Figure S4. Growth curves of *E. coli* MG1655 with fatty acids at 25 °C in LB medium. Bacteria were cultured to OD=0.1, and then butyric acid (**A**), caproic acid (**C**) and caprylic acid (**E**) were added at appropriate concentrations. Panels B,D and F show the optical density value of the culture at the end of the experiment: butyric acid (**B**), caproic acid (**D**) and caprylic acid (**F**). Statistical significance ($p<0.05$) is marked with an asterisk (*).

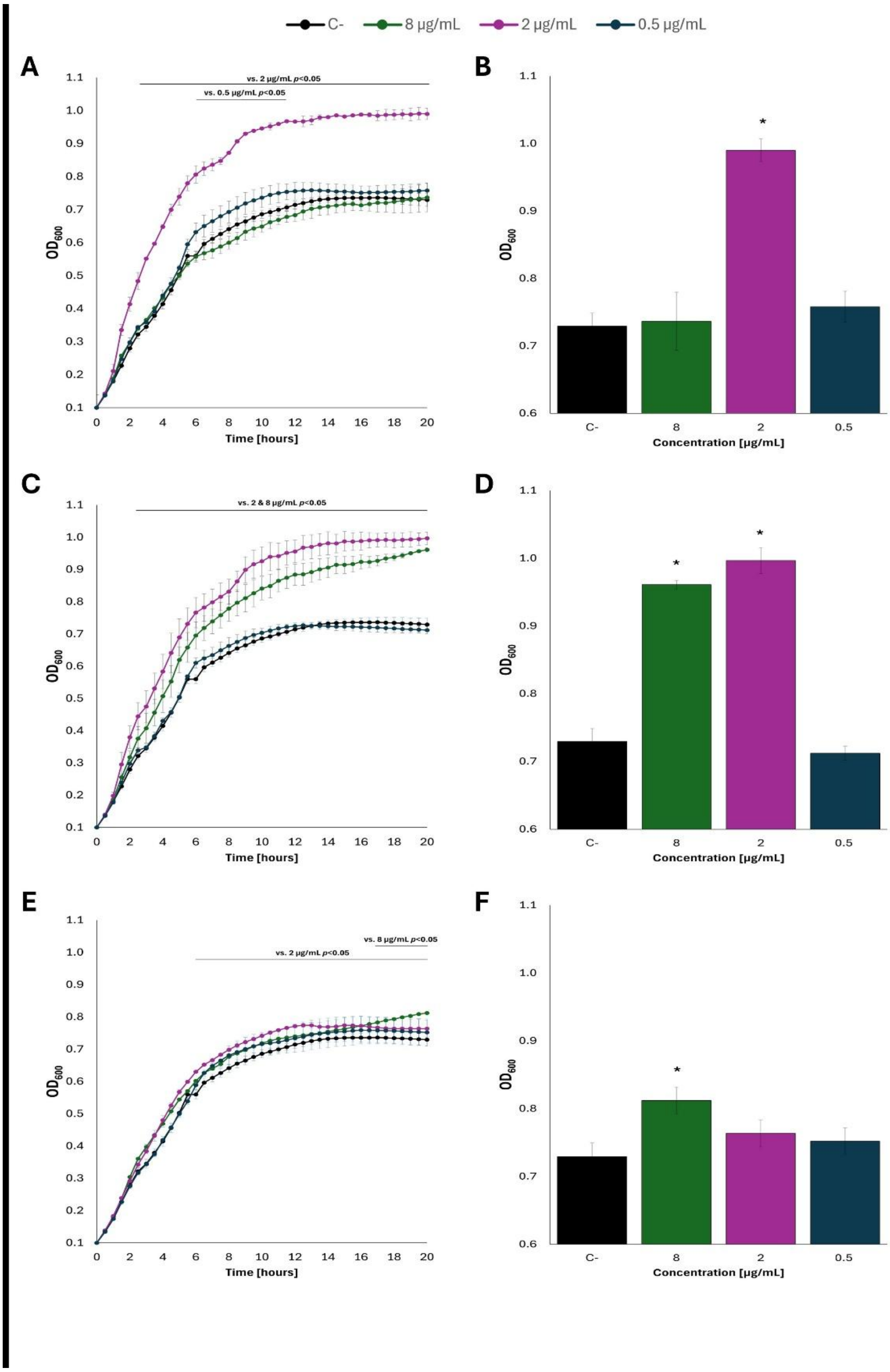

Figure S5. Growth curves of *E. coli* MG1655 with fatty acids (additional set of concentrations) at 25 °C in LB medium. Bacteria were cultured to OD=0.1, and then butyric acid (A), caproic acid (C) and caprylic acid (E) were added at appropriate concentrations. Panels B, D and F show the optical density value of the culture at the end the experiment: butyric acid (B), caproic acid (D) and caprylic acid (F). Statistical significance (p<0.05) is marked with an asterisk (*).

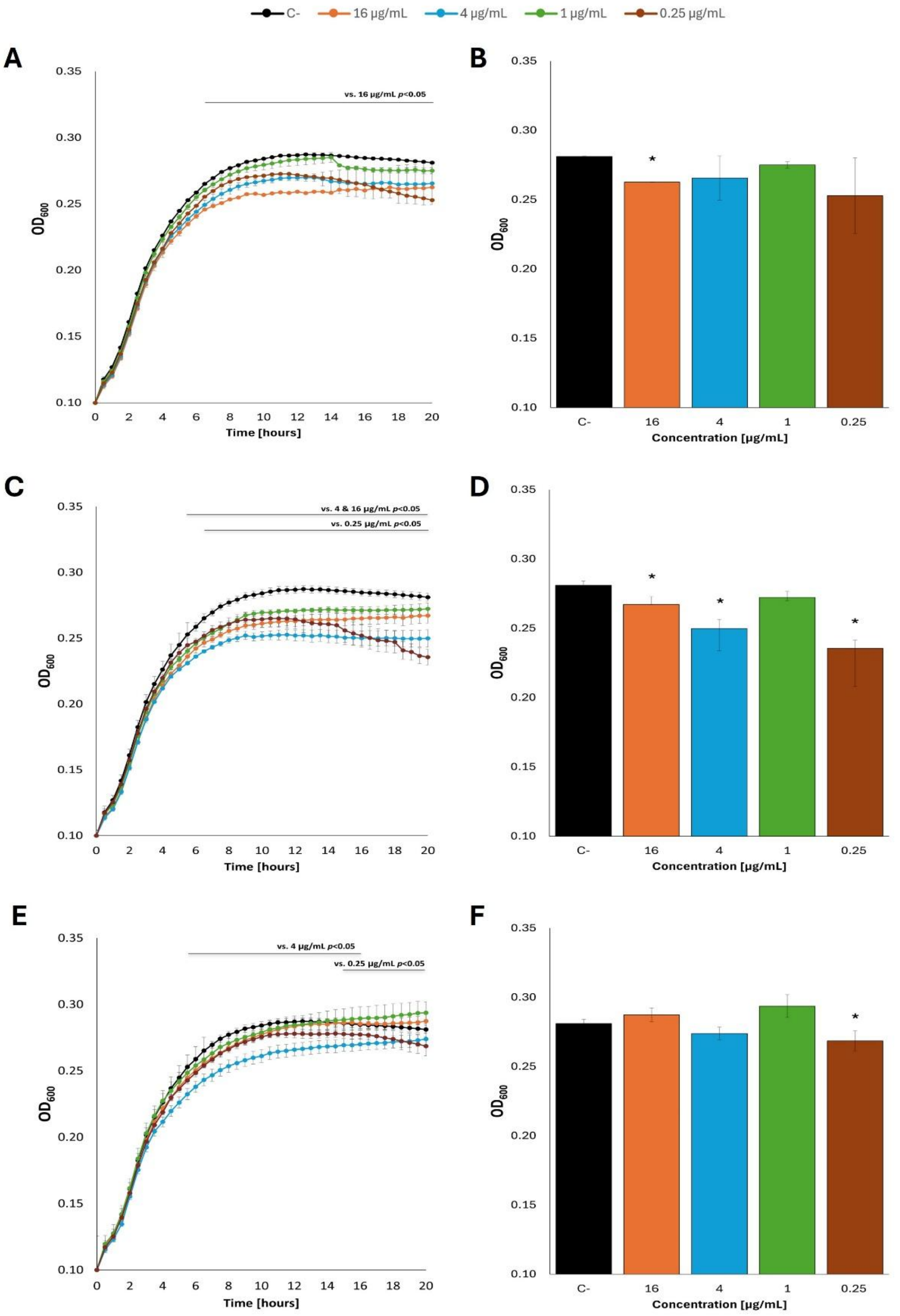

Figure S6. Growth curves of *E. coli* MG1655 with fatty acids at 37 °C in M9 minimal medium, in which glucose was the carbon source. Bacteria were cultured to OD=0.1, and then butyric acid (**A**), caproic acid (**C**) and caprylic acid (**E**) were added at appropriate concentrations. Panels B,D and F show the optical density value of the culture at the end of the experiment: butyric acid (**B**), caproic acid (**D**) and caprylic acid (**F**). Statistical significance ($p<0.05$) is marked with an asterisk (*).

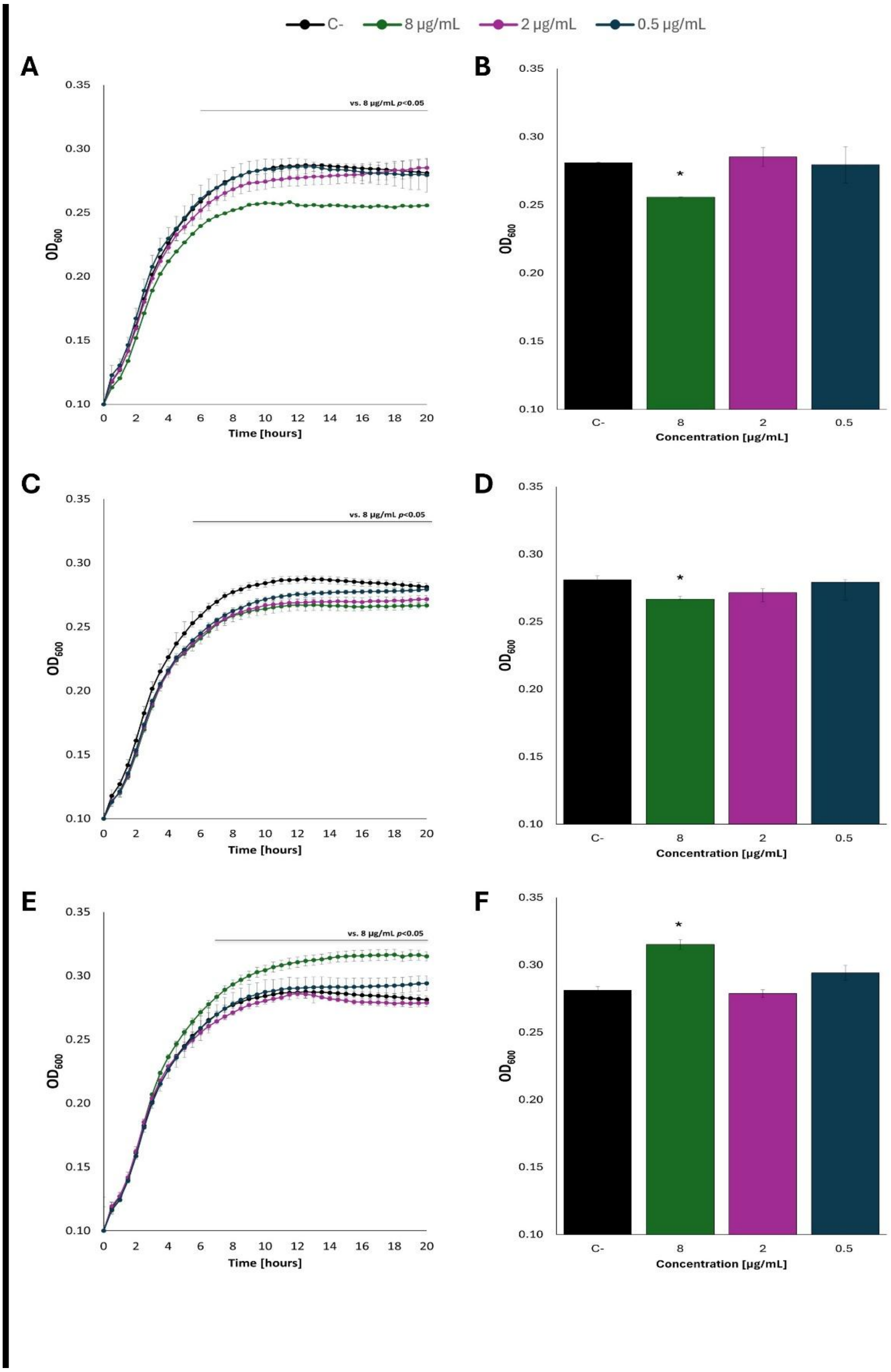

Figure S7. Growth curves of *E. coli* MG1655 with fatty acids (additional set of concentrations) at 37 °C in M9 minimal medium, in which glucose was the carbon source. Bacteria were cultured to OD=0.1, and then butyric acid (**A**), caproic acid (**C**) and caprylic acid (**E**) were added at appropriate concentrations. Panels B, D and F show the optical density value of the culture at the end of the experiment: butyric acid (**B**), caproic acid (**D**) and caprylic acid (**F**). Statistical significance (p<0.05) is marked with an asterisk (*).

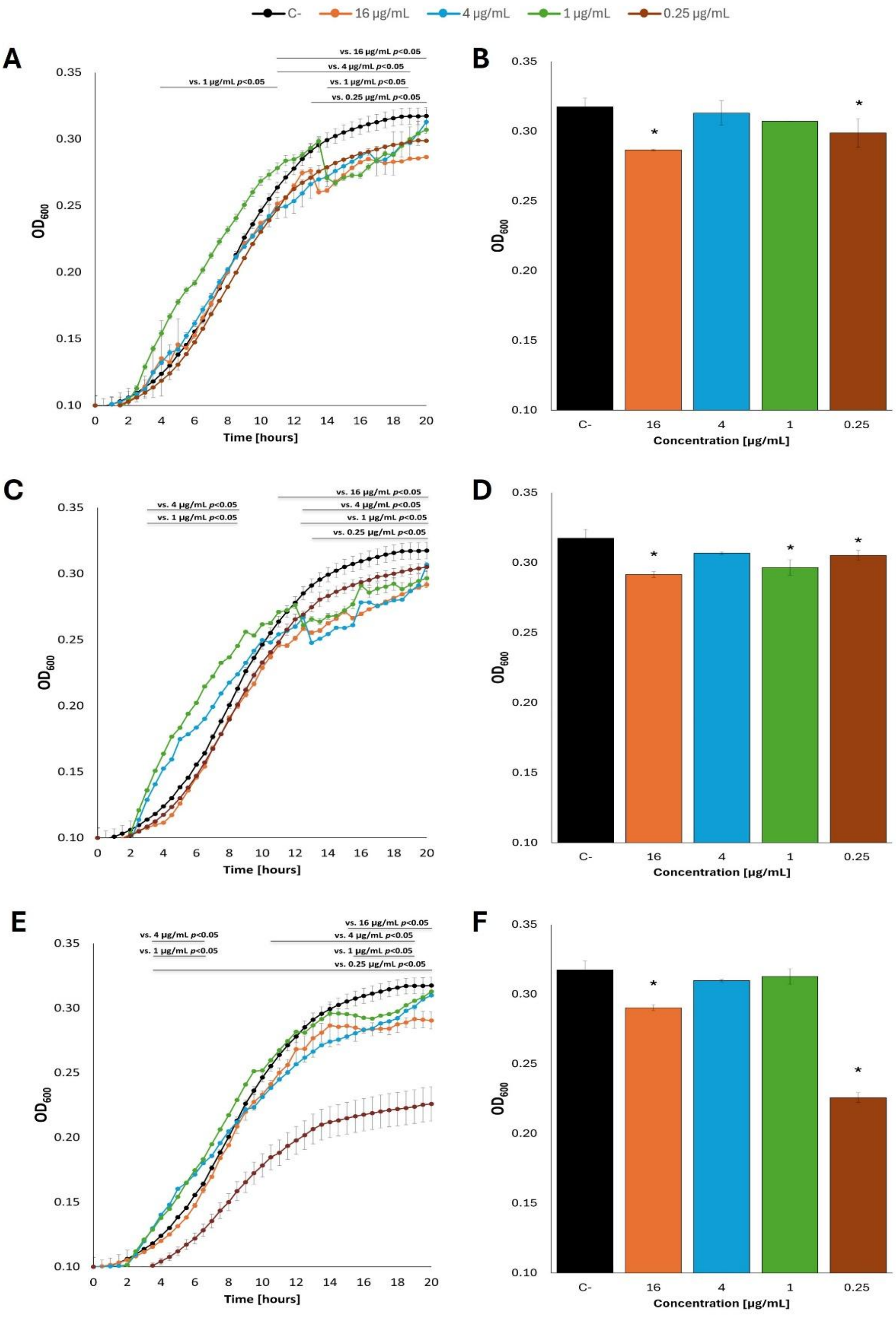

Figure S8. Growth curves of *E. coli* MG1655 with fatty acids at 25 °C in M9 minimal medium, in which glucose was the carbon source. Bacteria were cultured to OD=0.1, and then butyric acid (**A**), caproic acid (**C**) and caprylic acid (**E**) were added at appropriate concentrations. Panels B,D and F show the optical density value of the culture at the end of the experiment: butyric acid (**B**), caproic acid (**D**) and caprylic acid (**F**). Statistical significance ($p<0.05$) is marked with an asterisk (*).

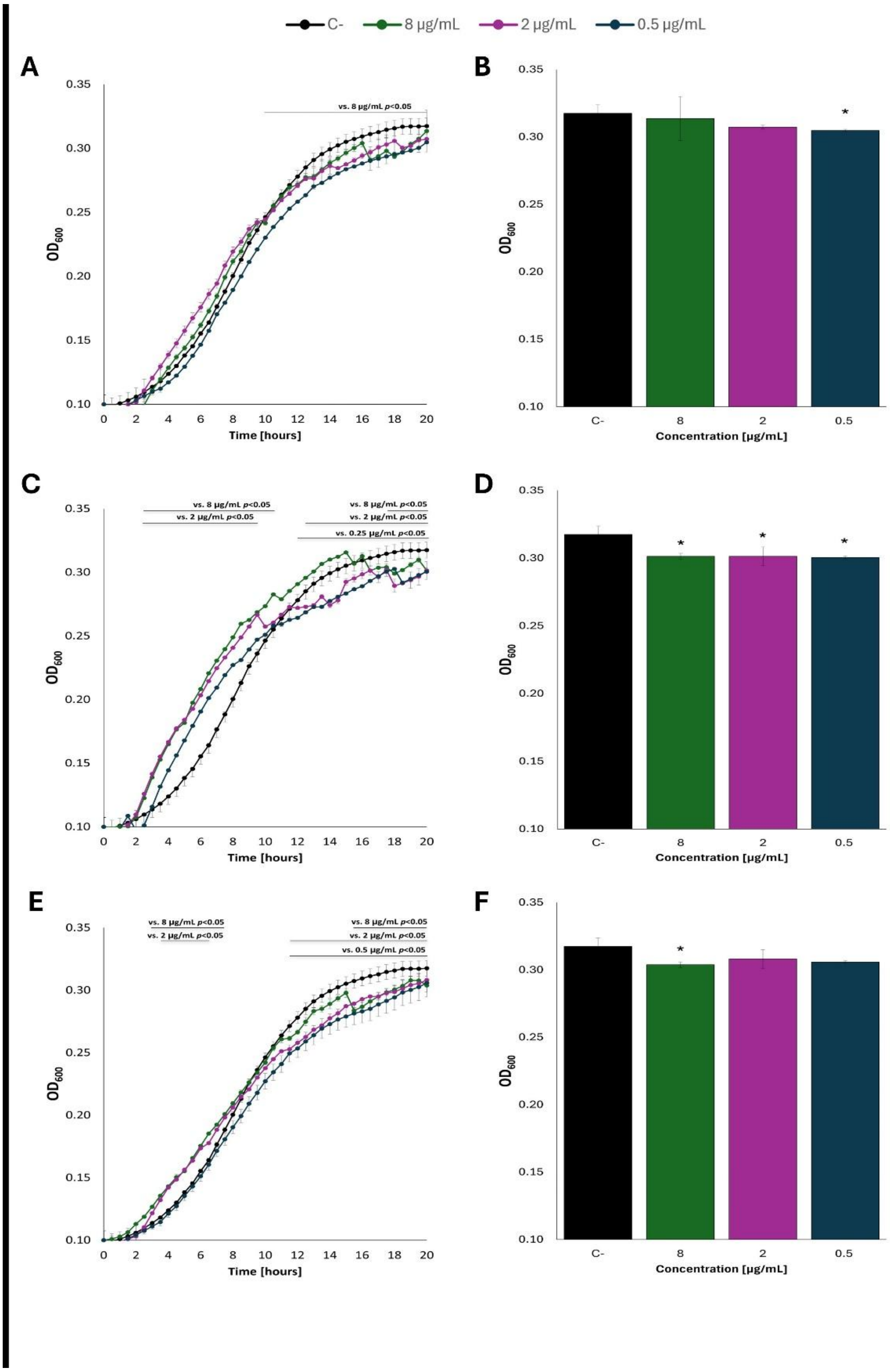

Figure S9. Growth curves of *E. coli* MG1655 with fatty acids (additional set of concentrations) at 25 °C in M9 minimal medium, in which glucose was the carbon source. Bacteria were cultured to OD=0.1, and then butyric acid (A), caproic acid (C) and caprylic acid (E) were added at appropriate concentrations. Panels B, D and F show the optical density value of the culture at the end of the experiment: butyric acid (B), caproic acid (D) and caprylic acid (F). Statistical significance ($p<0.05$) is marked with an asterisk (*).

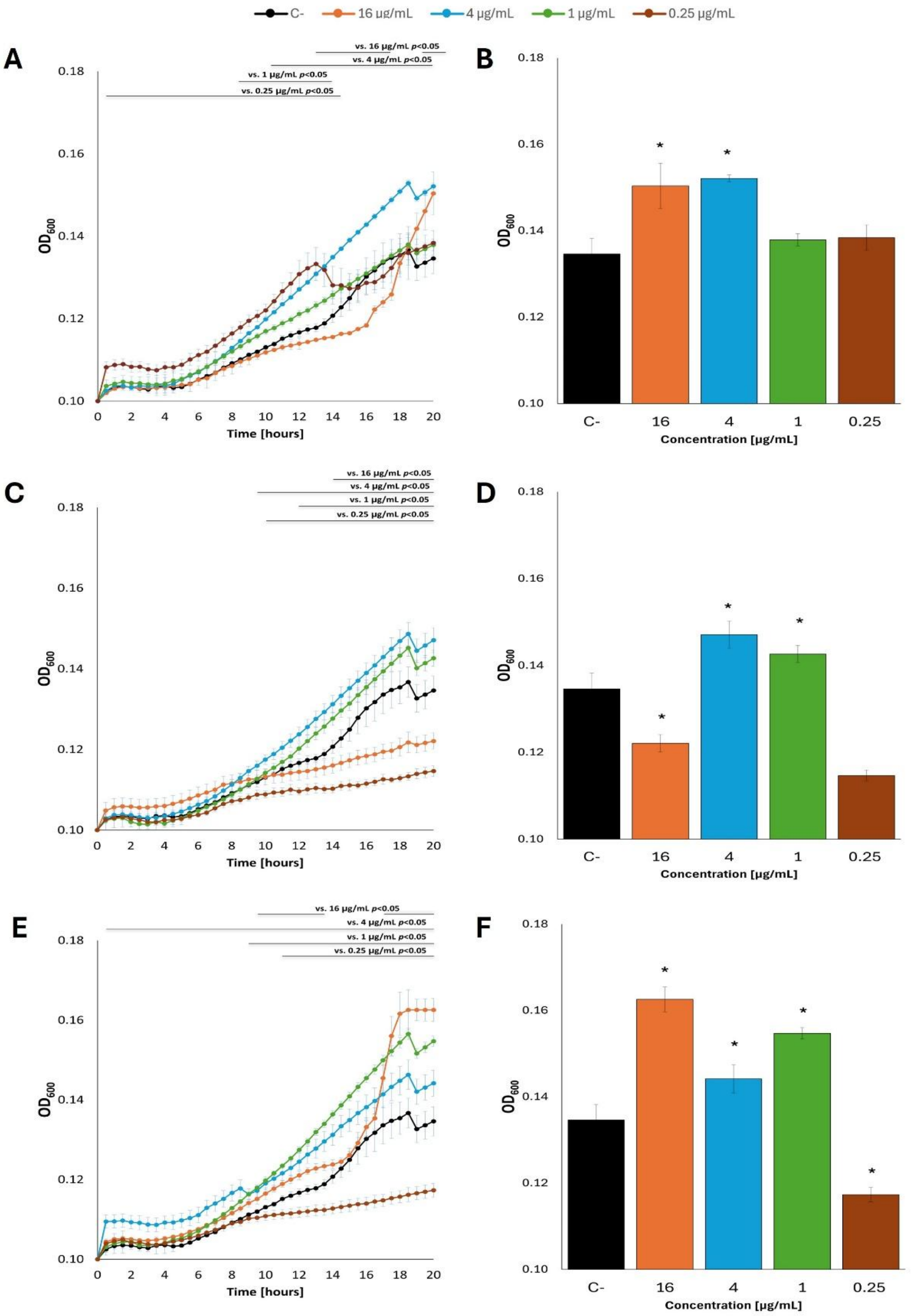

Figure S10. Growth curves of *E. coli* MG1655 with fatty acids at 37 °C in M9 minimal medium, in which acetate was the carbon source. Bacteria were cultured to OD=0.1, and then butyric acid (**A**), caproic acid (**C**) and caprylic acid (**E**) were added at appropriate concentrations. Panels B,D and F show the optical density value of the culture at the end of the experiment: butyric acid (**B**), caproic acid (**D**) and caprylic acid (**F**). Statistical significance (p<0.05) is marked with an asterisk (*).

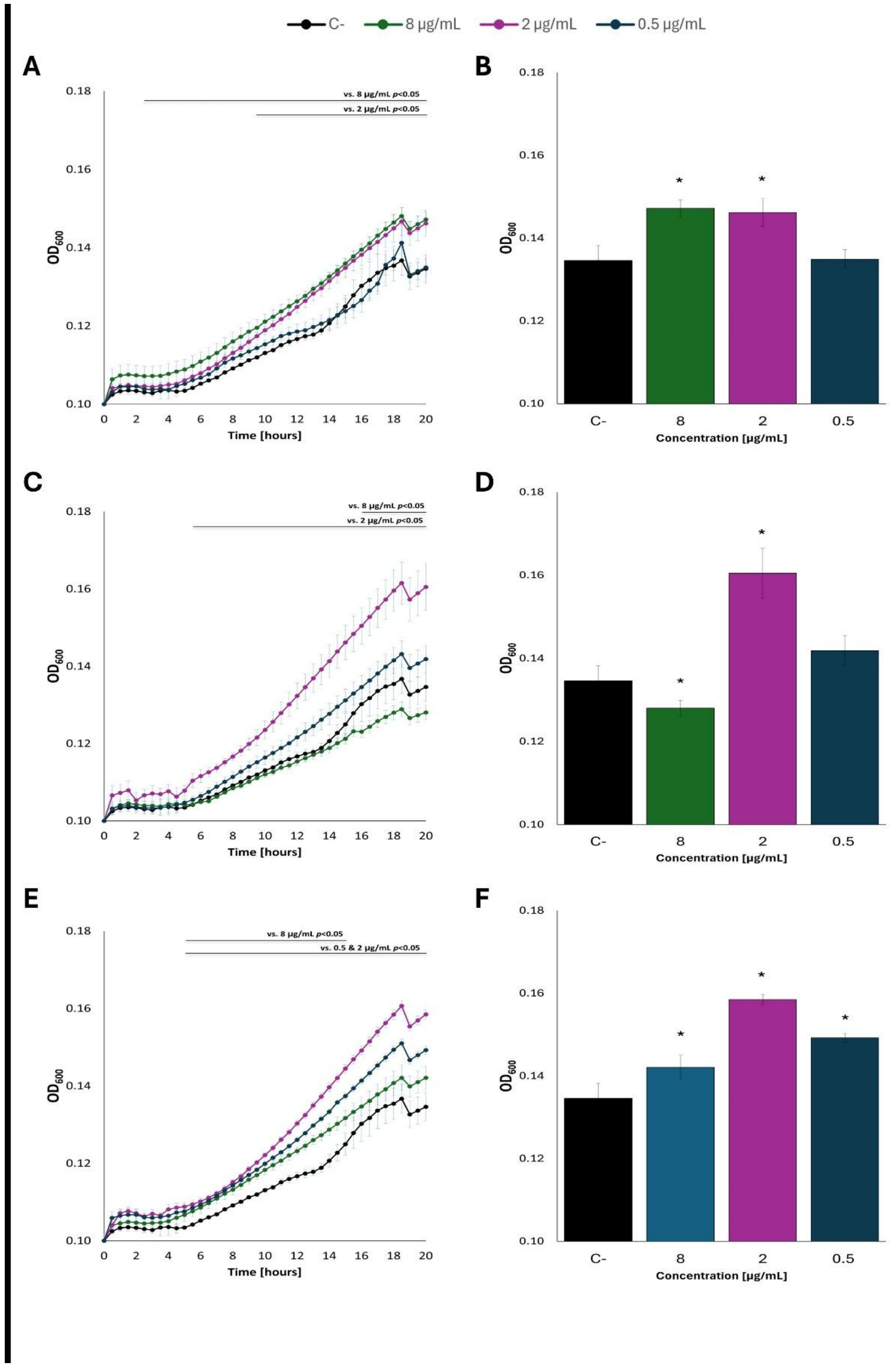

Figure S11. Growth curves of *E. coli* MG1655 with fatty acids (additional set of concentrations) at 37 °C in M9 minimal medium, in which acetate was the carbon source. Bacteria were cultured to OD=0.1, and then butyric acid (A), caproic acid (C) and caprylic acid (E) were added at appropriate concentrations. Panels B, D and F show the optical density value of the culture at the end of the experiment: butyric acid (B), caproic acid (D) and caprylic acid (F). Statistical significance ($p<0.05$) is marked with an asterisk (*).

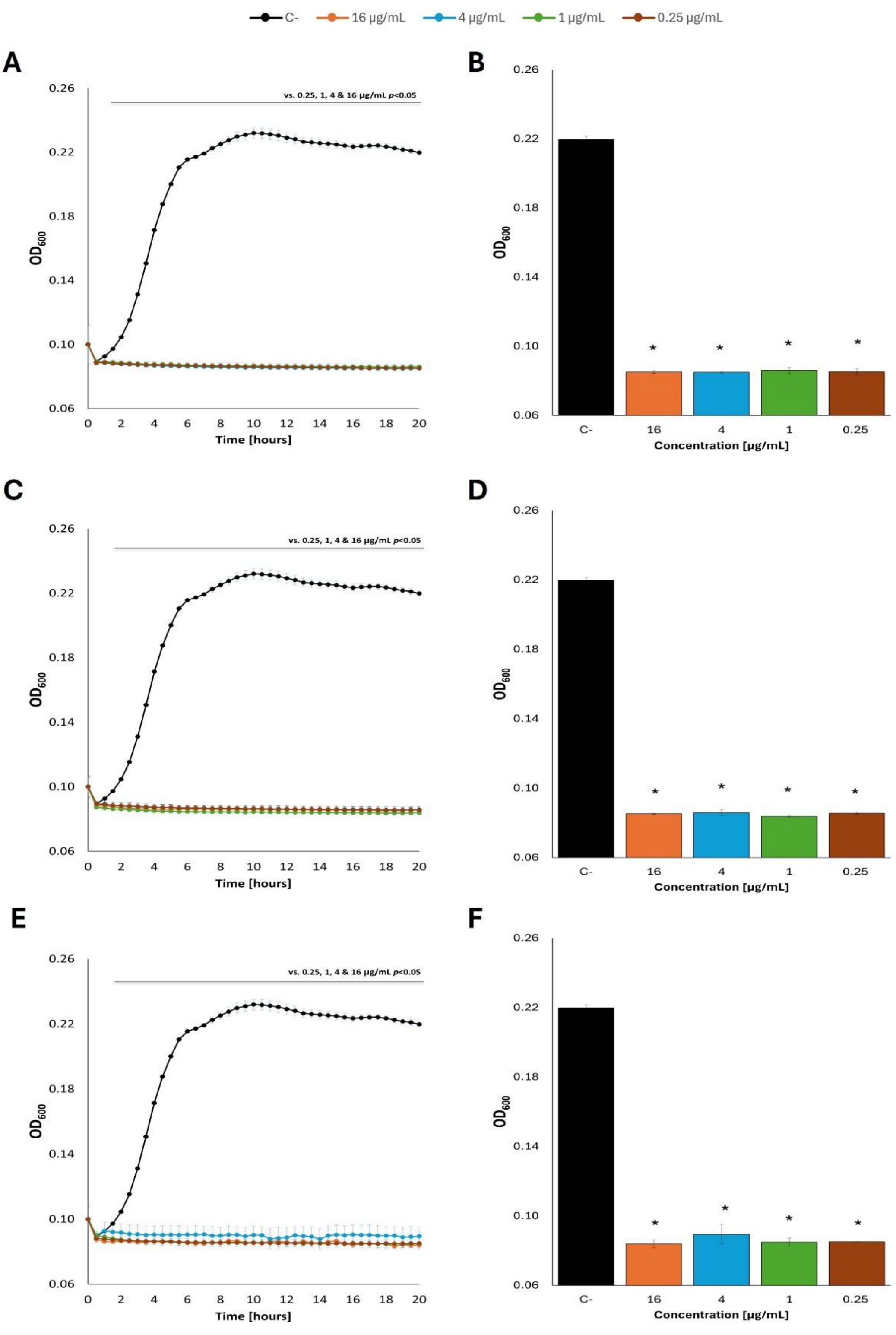

Figure S12. Growth curves of *E. coli* MG1655 with the addition of fatty acids at 37 °C in M9 minimal medium, in which glucose was the carbon source. The bacteria were cultured to OD=0.1, and then the previous carbon source was removed and replaced with: butyric acid (A), caproic acid (C) and caprylic acid (C) at appropriate concentrations. Panels B,D and F show the optical density value of the culture at the end of the experiment: butyric acid (B), caproic acid (D) and caprylic acid (F). Statistical significance (p<0.05) is marked with an asterisk (*).

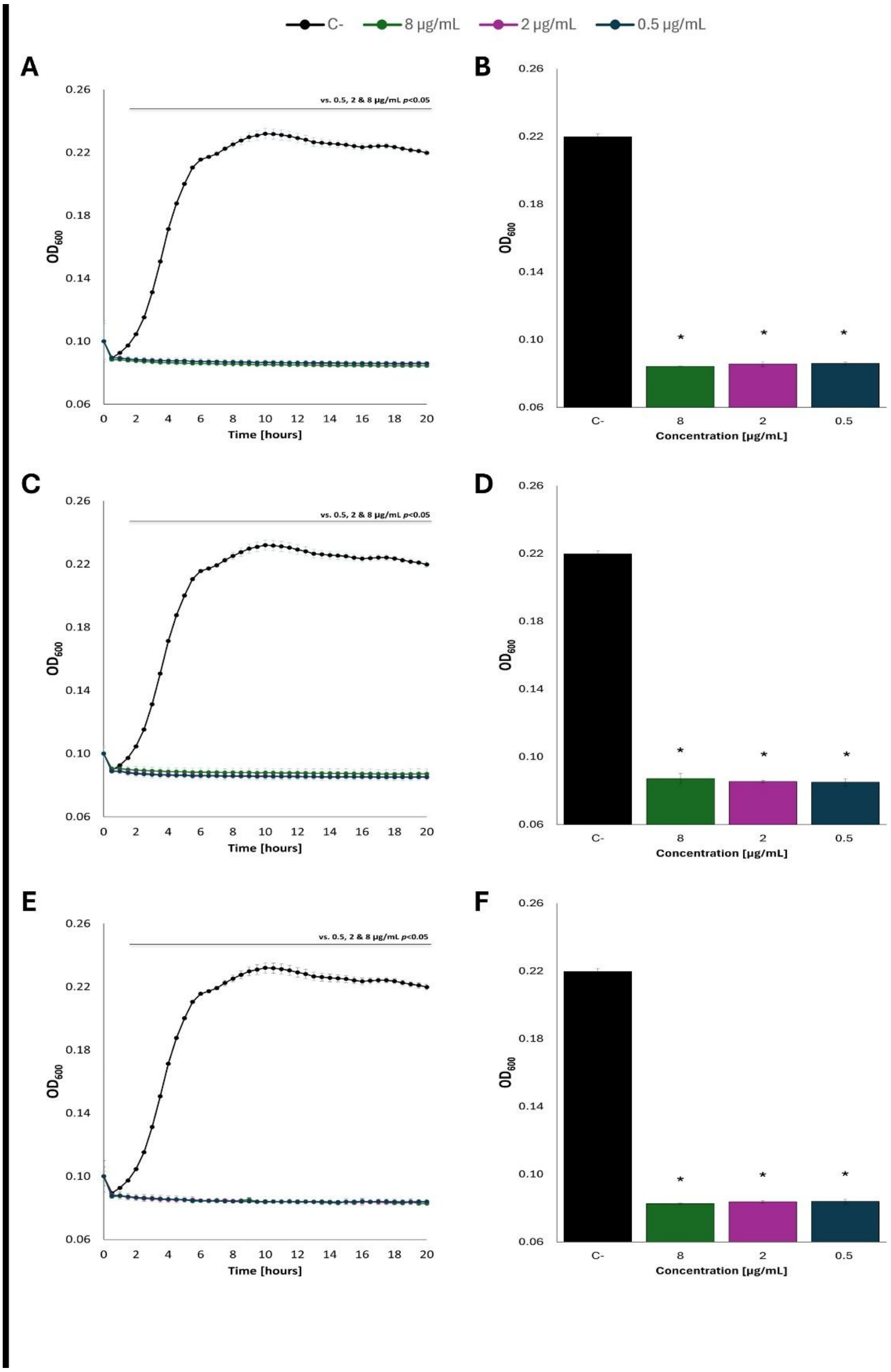

Figure S13. Growth curves of *E. coli* MG1655 with the addition of fatty acids (additional set of concentrations) at 37 °C in M9 minimal medium, in which glucose was the carbon source. The bacteria were cultured to OD=0.1, and then the previous carbon source was removed and replaced with: butyric acid (A), caproic acid (C) and caprylic acid (C) at appropriate concentrations. Panels B, D and F show the optical density value of the culture at the end of the experiment: butyric acid (B), caproic acid (D) and caprylic acid (F). Statistical significance (p<0.05) is marked with an asterisk (*).

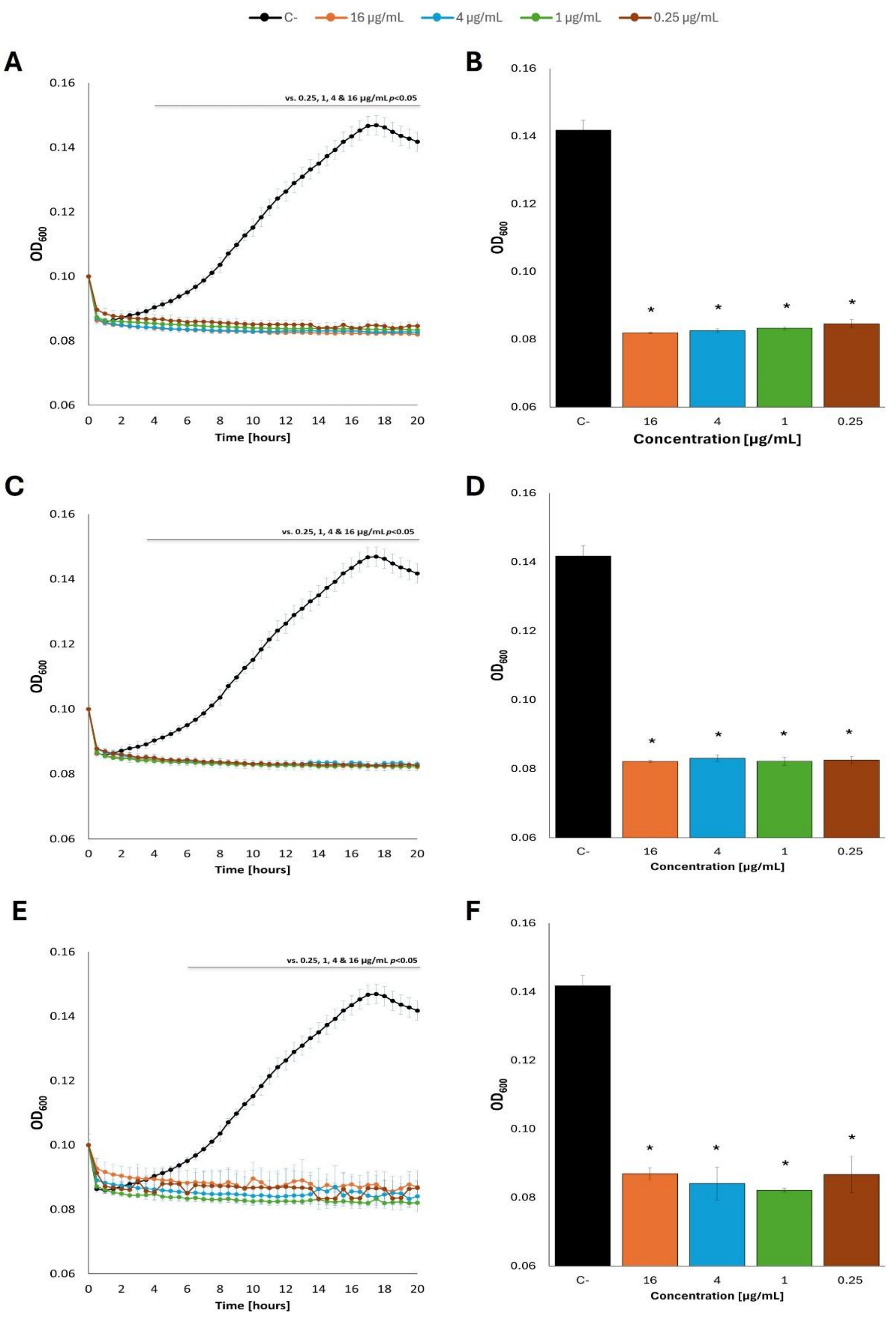

Figure S14. Growth curves of *E. coli* MG1655 with the addition of fatty acids at 37 °C in M9 minimal medium, in which acetate was the carbon source. The bacteria were cultured to OD=0.1, and then the previous carbon source was removed and replaced with: butyric acid (A), caproic acid (C) and caprylic acid (C) at appropriate concentrations. Panels B,D and F show the optical density value of the culture at the end of the experiment: butyric acid (B), caproic acid (D) and caprylic acid (F). Statistical significance (p<0.05) is marked with an asterisk (*).

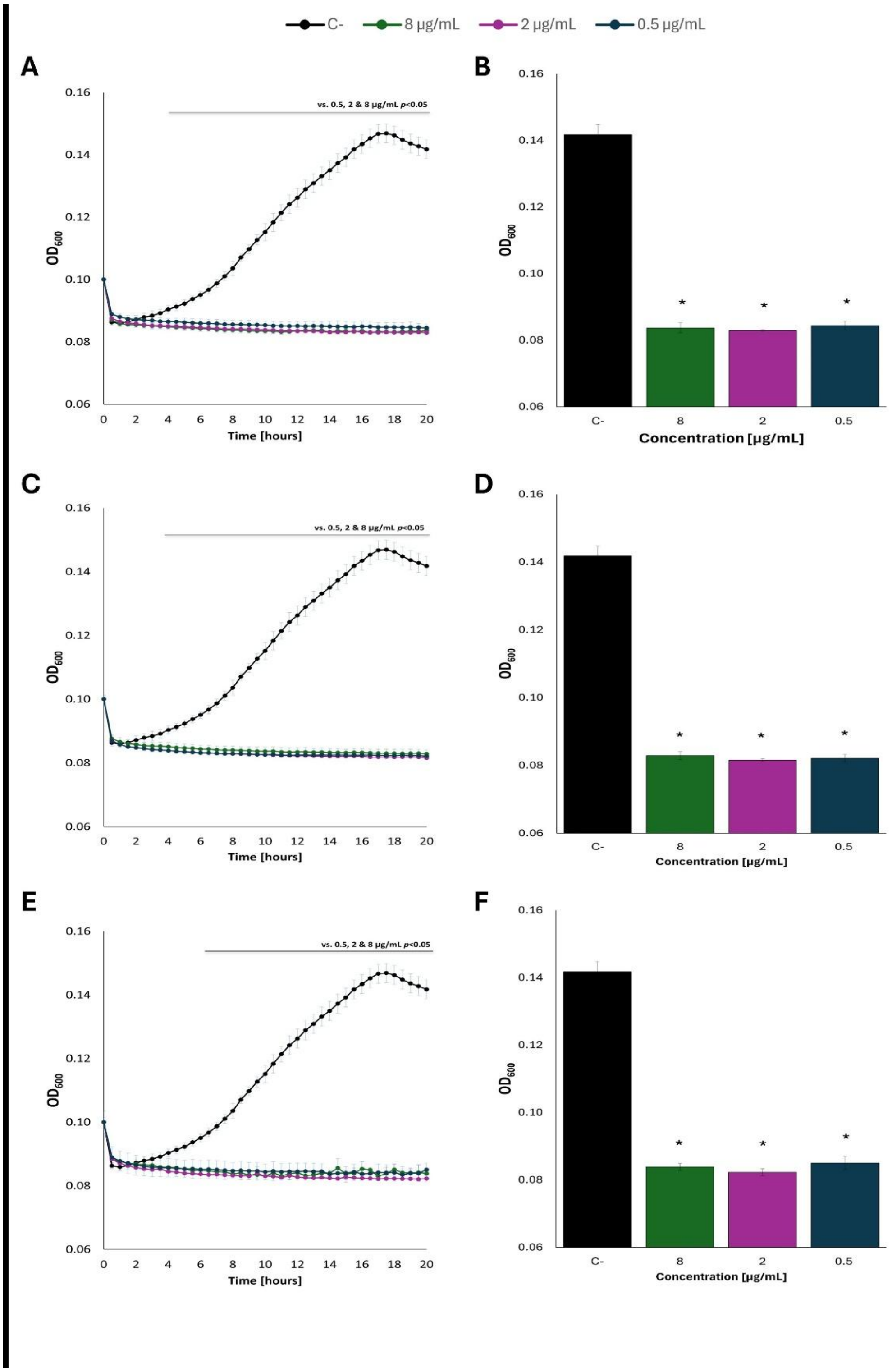

Figure S15. Growth curves of *E. coli* MG1655 with the addition of fatty acids (additional set of concentrations) at 37 °C in M9 minimal medium, in which acetate was the carbon source. The bacteria were cultured to OD=0.1, and then the previous carbon source was removed and replaced with: butyric acid (A), caproic acid (C) and caprylic acid (C) at appropriate concentrations. Panels B, D and F show the optical density value of the culture at the end of the experiment: butyric acid (B), caproic acid (D) and caprylic acid (F). Statistical significance (p<0.05) is marked with an asterisk (*).
